# Increased Stopover Duration and Low Body Condition of the Pied Flycatcher (*Ficedula hypoleuca*) at an Autumn Stopover Site

**DOI:** 10.3390/ani10122208

**Published:** 2020-11-25

**Authors:** Bernice Goffin, Marcial Felgueiras, Anouschka R. Hof

**Affiliations:** 1 Wildlife Ecology and Conservation Group, Wageningen University, 6708 PB Wageningen, The Netherlands; Anouschka.Hof@wur.nl; 2 A Rocha, Apartado 41, 8501-903 Mexilhoeira Grande, Portugal; Marcial.Felgueiras@arocha.org; 3 Department of Wildlife, Fish, and Environmental Studies, Swedish University of Agricultural Sciences, 901 87 Umeå, Sweden

**Keywords:** climate change, migration strategy, movement ecology, passerines

## Abstract

**Simple Summary:**

Many bird species that migrate long distances are in decline partly because of environmental changes, such as climate change or land-use changes. Although much is already known on the effects of environmental change on birds that are on their spring migration or on their breeding grounds, little is known with regard to possible negative effects on birds that are on their autumn migration and visiting so-called stopover sites on their way to their wintering grounds. These stopover sites are vital for birds to refuel, and a potential deteriorating quality of the stopover sites may lead to individuals dying during migration. We investigated the impacts of local environmental conditions on the migration strategy and body condition of the Pied Flycatcher at an autumn migration stopover site using long-term ringing data and local environmental conditions. We found that although birds arrived and departed the stopover site around the same time over the years, the body condition of the individuals caught decreased, and the length of their stay at the stopover site increased. This suggests that conditions at the stopover site during the autumn migration period have deteriorated over time which may lead to the death of more birds during autumn migration.

**Abstract:**

Many long-distance migratory bird species are in decline, of which environmental changes, such as climate change and land-use changes, are thought to be important drivers. The effects of environmental change on the migration of these birds have often been studied during spring migration. Fewer studies have explored the impacts of environmental change on autumn migration, especially at stopover sites. However, stopover sites are important, as the quality of these sites is expected to change over time. We investigated impacts of local environmental conditions on the migration strategy and body condition of the Pied Flycatcher (*Ficedula hypoleuca*) at an autumn migration stopover site using long-term ringing data (1996–2018) and local environmental conditions. We found that although the arrival and departure dates of birds at the stopover site remained unchanged, the body condition (fat score) of the individuals caught decreased, and the stopover duration increased. This suggests that conditions at the stopover site during the autumn migration period have deteriorated over time. This study emphasizes the importance of suitable stopover sites for migratory birds and stresses that changes in environmental conditions during the autumn migration period may be contributing to the current decline in long-distance migratory passerines.

## 1. Introduction

Migration is a common strategy in birds that have to deal with seasonal variation in resource availability. By migrating to temperate regions in spring, birds can exploit the high food availability there to raise offspring as well as avoid the higher nest predation risk in tropical regions [1,2,3]. However, migration is also a risky strategy; it has been shown for several bird species, especially for passerines, that mortality is relatively high during migration [4,5,6,7]. Migration costs a lot of energy, and birds need to accumulate a large amount of fat reserves in order to complete their migration successfully [8]. Therefore, during migration, many bird species will regularly stop over at suitable sites in order to replenish these fat reserves [9,10,11]. Although studies on mortality at stopover sites are scarce, it is believed that the quality of stopover sites is an important factor in determining the survival rate of migrating birds [6,12].

In recent decades, many migratory species have been declining in abundance [13,14], and environmental change is thought to pose an extra threat to these species [15]. First of all, it is expected that long-distance migrants will have to deal with increasing competition from non-migrating birds that are benefitted by warmer winters in temperate areas [16]. Furthermore, earlier onset of spring in temperate areas causes the birds to arrive too late at the breeding sites, creating a mismatch between the time of peak food demand of chicks and time of peak food availability [17]. This mismatch has been identified as a major cause of the decline in the reproductive success of migratory birds [17]. Studies investigating changes in bird migration in relation to environmental change have often focused on spring migration and generally agree that the arrival time at the breeding grounds has advanced over time and that conditions at and locations of wintering grounds can have large effects on timings of spring migration [18,19,20,21,22,23]. Less literature is available on the effects of environmental change on autumn migration, although it has been shown in several studies that long-distance European migrants that migrate across the Sahara Desert have advanced their autumn migration [24,25]. A probable cause for this advancement in autumn migration is the earlier dry season in the Sahel, which makes it beneficial for birds to depart to their wintering grounds earlier [25]. It has further been suggested that a climate change-induced advancement of breeding may affect the timing of autumn migration due to an increased frequency in second broods [26]. Even less literature is available on the effects of environmental change on stopover sites of long-distance migrants, even though the timing of autumn migration and quality of stopover sites are of great importance to migratory bird survival [6,25]. To fully understand the impacts that environmental change may have on long-distance migratory birds, impacts of environmental change on stopover sites should also be investigated. As many long-distance migratory birds are of special conservation concern [14,27], identifying possible threats to this relatively vulnerable bird group is essential in order to improve conservation measures.

Here, we investigated the impacts of local climate variation on the migration strategy of the Pied Flycatcher (*Ficedula hypoleuca*) at an important stopover site in southern Portugal, which is used by many birds to refuel before the ocean gap between South Iberia and the Moroccan coast following continuation on to the Sahara [28,29]. This small insectivorous passerine, which breeds in large parts of Europe and western Asia and winters in sub-Saharan Africa [30], has already been studied extensively as a model species of a long-distance migratory passerine. Similar to many other long-distance migrants, the Pied Flycatcher population is declining [30]. This decline has earlier been attributed to mistimed reproduction in spring as a consequence of an earlier food peak [15,31]. Thus far, possible impacts of climate-related changes in autumn stopover sites on Pied Flycatchers have not been studied to our knowledge.

Southern Portugal has experienced an increase in temperature and decrease in precipitation during spring over the past few decades, causing drier conditions throughout the summer [32,33]. These drier conditions have been linked to an increase in fires throughout the region, which have a detrimental effect on woodland habitat [34,35]. As woodlands are the preferred habitat of the Pied Flycatcher [30], the species is expected to suffer from considerable habitat loss and decreased food availability at its stopover sites in the Mediterranean region. This may have large consequences for survival rates of this species and others that rely on similar habitat types.

We used a long-term ringing database of an autumn stopover site in the Algarve region in southern Portugal to assess the impacts of local climate variation on the autumn migration strategy and body condition of the Pied Flycatcher. We expected Pied Flycatchers to advance their autumn migration and thus advance their arrival at the studied stopover site in accordance with earlier studies on autumn migration timing in long-distance European migrants [24,25]. Moreover, we expected the body condition of Pied Flycatchers at the stopover site to decrease over time due to worsening environmental conditions at the site [32,33,34,35]. Therefore, we also expected to find an increase in stopover duration of the birds at the stopover site, as they will need more time to increase their body condition enough for a successful continuation of their migration.

## 2. Materials and Methods

### 2.1. Data Collection

We used a long-term ringing database (1996–2018) collected on the grounds of Cruzinha (9660 m^2^; coordinates 37°08′39.8″ N, 8°36′29.2″ W), which is the research center of the nature conservation organization A ROCHA Portugal (https://arocha.pt/en/). This center is located in the Algarve region in southern Portugal, in a rural area about two kilometers south of the town Mexilhoeira Grande. The Cruzinha grounds consist of shrubland, a pond with a reedbed, a wooded area with tall pine trees, and an orchard and vegetable garden. This variation in habitats leads to a large variation in bird species visiting the grounds to refuel, including the Pied Flycatcher. The surrounding area is dominated by abandoned farmland, scrubland, and dry orchards.

Birds have been captured with mist nets and ringed on the study site since 1987. However, catching effort has only been standardized since 1996. Therefore, we only used data from the period 1996–2018, and for one aspect of the study from the years 2005–2018, for the analyses. In most cases, 10 mist nets, which sum up to a total net length of 147 m, were opened during days on which birds were ringed. Although bird ringing was done at least once a week, it was sometimes done more often. Especially during the autumn migration, when an influx in birds passing through was expected, the ringing activity was high. However, catching effort was always recorded and could be accounted for in the analyses (see below). A total of 71 bird ringers have gathered Pied Flycatcher data since 1996. Bird ringing was almost always done in the morning, although rare exceptions are present. Many different bird species were caught at the site, but in this study, we only focused on captures of the Pied Flycatcher, it being one of the most frequently caught birds during autumn migration. The Pied Flycatcher does not breed in the area [30], and thus, almost all Pied Flycatchers were caught during their autumn migration in the months of September and October, although some individuals were also caught in July, August, and November. Individuals were sometimes also caught in April and May, but these individuals were assumed to be on their spring migration instead of on their autumn migration and therefore have been excluded from the analyses. In addition, same day recaptures of individuals were excluded from the analyses.

Capture date and time was recorded for every bird caught in the mist nets. Birds that had been caught for the first time received an aluminium ring with a unique code for individual recognition. Maximum chord wing length in millimeters, mass in grams using an electronic scale, and the fat score according to the methods of Kaiser [36] were measured. Moreover, the age of the Pied Flycatchers was determined as either first calendar year or older than first calendar year by the pattern on the central tertial [37]. In addition, when possible, the sex was determined based on plumage colouration [37]. If a bird had been captured that had an aluminum ring already, the ring number was recorded. The rest of the measurements on birds that were at least recaptured once during their stay were done as usual, except for the wing length, which was not measured upon recapture.

The number of and the amount of time nets were opened varied as well as the number of days ringing took place per year. Therefore, we corrected for catching effort when calculating the annual total amount of individuals captured. On every day that bird ringing took place, it has been recorded when the nets were opened and which nets were opened. The closing time of the nets was not always specified in the database. Therefore, in order to approach the closing time of the nets as closely as possible, the time of the last capture of the day was recorded as the closing time of the nets. Then, the amount of hours that the nets were open, rounded to quarter hours, could be calculated for each day. As the length of every net in meters is known, the total amount of net meters could also be calculated per day. Then, the catching effort per day was determined by multiplying the amount of net meters with the amount of hours that the nets were open that day. Yearly catching effort was calculated by summing all the values of daily catching effort within the autumn migration period of the Pied Flycatcher in that year. For this calculation, the autumn migration period of the Pied Flycatcher per year was determined by taking the time between the date of first capture and date of last capture of a Pied Flycatcher in the period July–November.

Several variables concerning Pied Flycatcher migration strategy and body condition have subsequently been extracted from this effort-based ringing dataset (Table 1). All averages in Table 1 were calculated per year. The annual total amount of individuals captured was corrected with the yearly catching effort by dividing the annual total amount of individuals by the yearly catching effort. The average arrival date and departure date were specified as the average capture date of respectively the first five percent and the last five percent of individuals. The minimum stopover duration was calculated by subtracting the date that the individual was captured for the first time from the date that the individual was captured for the last time plus one. This “one” was added because Pied Flycatchers migrate at night and therefore are not assumed to leave the area on the day that they are captured [29]. Minimum stopover duration can be a useful way to measure the true stopover duration when other variables are also measured that are based on the first and last capture of an individual [38]. As this is the case for our study, minimum stopover duration was used as a measure of stopover duration. Years in which only one or two birds were recaptured were not used for the recapture-based analyses.

In order to relate changes in the migration strategies and body condition of the Pied Flycatchers to local environmental conditions, two variables related to the climate were taken into account (Table 1). All local climate data used for this study were based on data obtained at a weather station present at the study site. At this weather station, the average temperature (based on maximum and minimum temperature) and precipitation were measured every day from January 2005 onwards. Temperatures were measured in degrees Celsius while rounding off on a single decimal, and the precipitation was measured in millimeters, rounding off on each half millimeter. The autumn migration period was defined as the period between the average arrival date and the average departure date where the arrival date and departure date were averaged across the period 2005–2018 in order to keep the defined autumn migration period constant each year. Equally detailed local weather data before 2005 were unfortunately not available to us. Since the weather data were collected daily at the ringing site, we opted to use these weather data rather than coarser data from a weather station located further from the ringing site. Therefore, these analyses were only based on the years 2005–2018.

### 2.2. Statistical Analyses

Linear models were used to uncover possible trends in the migration strategy of the Pied Flycatchers over the years. Analyses were based on data from 1996 until 2018. General linear models were used to assess the impact of local climate on the migration strategy of Pied Flycatchers. As local climate variables were only available from 2005 onwards, these analyses were based on data from 2005 until 2018. All analyses were conducted in R version 3.5.0 [39], using the ‘lme4’ package [40]. The environmental variables were tested for multicollinearity using a Variance Inflation factor (VIF) test from the ‘car’ package [41]. Multicollinearity was assumed if the Variance Inflation factor (VIF) value of at least one of these variables was higher than five [42,43]. As this was not the case for any of the environmental variables, multicollinearity was not assumed, and all of the environmental variables were incorporated in the models. The assumptions of the models were verified using the ‘DHARMa’ package [44]. For the model with the response variable “minimum stopover duration”, a log transformation was applied on the response variable in order to verify the assumptions. In addition, the assumptions of the variables “mass at arrival” and “fat gain speed” were not met even after transformation. Therefore, these variables were not included in the models. The assumptions of the remaining models could be verified without transformation. After the model assumptions had been verified, the models were checked for significant correlations. Then, all non-significant correlations were removed from the model until only significant correlations (*p* < 0.05) remained (backwards selection).

In the period 1996–2018, 70 recaptured individuals (same-day recaptures excluded) at the study site had both their sex and age recorded. In order to test for variation in migration strategy between sex and age groups, the individuals that were recaptured were classified in four different groups: young (first calendar year) males (*n* = 25), young females (*n* = 20), old (older than first calendar year) males (*n* = 8), and old females (*n* = 17). Differences between these groups in minimum stopover duration, mass gain, mass gain speed, mass at arrival, mass at departure, fat gain, fat gain speed, fat at arrival, and fat at departure were assessed. The variables “minimum stopover duration”, “mass gain speed”, “fat gain”, “fat gain speed”, “fat at arrival” and “fat at departure” did not follow a normal distribution (Shapiro–Wilk, all *p* > 0.05) and thus, differences within these variables were analyzed using a Kruskal–Wallis tests followed by a Wilcoxon Rank Sum test if a significant difference between different groups was found. The Shapiro–Wilk test for normality on the variable “mass gain” was very close to significance as well (*p* = 0.051). Due to this near-significant result in combination with the limited sample size of this analysis, it was decided to use the Kruskal–Wallis test and Wilcoxon Rank Sum test for this variable as well. The variables “mass at arrival” and “mass at departure” followed a normal distribution (Shapiro–Wilk, all *p* > 0.7), and thus, differences within these variables were analyzed using a one-way ANOVA followed by a Tukey HSD (honestly significant difference) test if a significant difference between groups was found.

## 3. Results

### 3.1. Migration Strategies

A total of 1290 Pied Flycatchers were captured between 1996 and 2018, containing 147 individuals that were recaptured at least once during their stay at the study site (same-day recaptures excluded) and thus were used for the recapture-based analyses (Table 1). Of these 147 recaptured individuals, only the 70 individuals that had both their sex and age recorded were used for the analyses between sex and age groups. For the analyses with local environmental variables, only the captures between 2005 and 2018 could be used due to the lack of weather data from before 2005. In this period, 859 Pied Flycatchers were captured in total, and 107 individuals were recaptured at least once during their stay. Analyses showed that there was no change in the number of individuals caught over the years (t = −1.27, df = 1, *p* = 0.22), and there was no significant relation between the number of individuals caught over the years and any of the local environmental variables (all *p* > 0.05).

**Figure 1:**
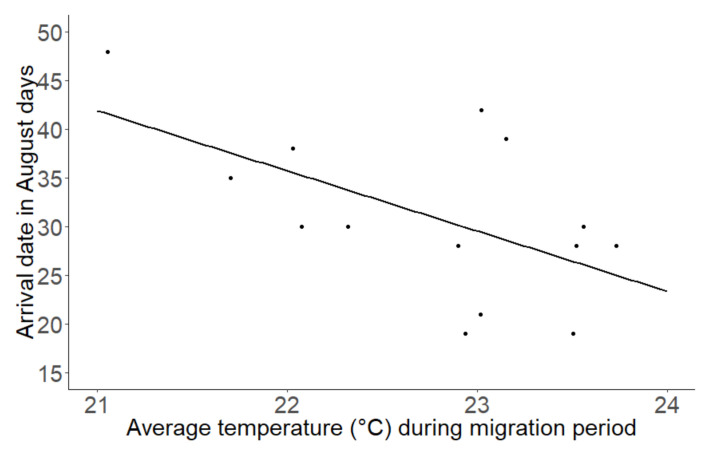
The arrival date (in August days) at the study site versus the average temperature at the stopover site during the autumn migration period during the period of 1996–2018.

Mean arrival date (mean = August 29th; SD = 9.5) and departure date (mean = October 16th, SD = 6.1) of Pied Flycatchers at the stopover site did not change significantly over the years (arrival date: t = −0.15, df = 1, *p* = 0.88, departure date: t = 0.03, df = 1, *p* = 0.98). We found a significant negative correlation between mean arrival date and “average temperature during the autumn migration period” (t = −2.44, df = 1, *p* = 0.031, Figure 1). This suggests that Pied Flycatchers arrived at the study site earlier when average temperatures were high at the stopover site. There was no significant relation between the departure date at the stopover site and any of the local environmental variables (all *p* > 0.05).

**Figure 2:**
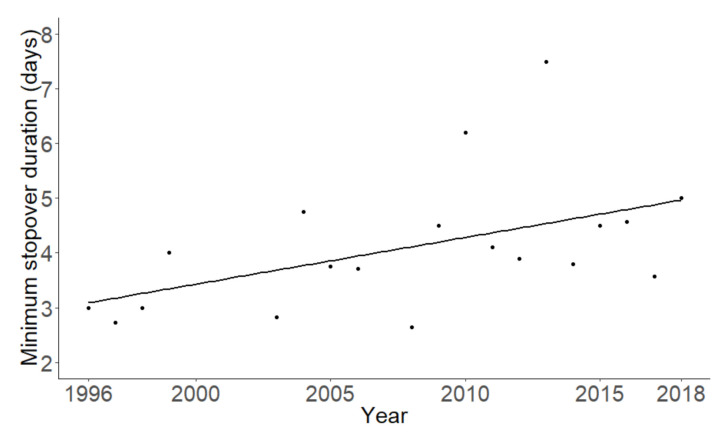
The average minimum stopover duration at the study site of Pied Flycatchers over the years.

The minimum stopover duration increased significantly over the years (t = 2.34, df = 1, *p* = 0.032, Figure 2). However, no significant correlations between minimum stopover duration and any of the environmental variables were found (all *p* > 0.05). Furthermore, minimum stopover duration did not significantly differ between different age and sex groups (Kruskal–Wallis, *x*^2^ = 6.02, df = 3, *p* = 0.11).

### 3.2. Body Condition

The average mass of Pied Flycatchers at the study site did not change significantly over the years (t = 0.95, df = 1, *p* = 0.35). Moreover, both mass gain and mass gain speed did not change significantly over the years (mass gain: t = 1.78, df = 1, *p* = 0.09, mass gain speed: t = 0.58, df = 1, *p* = 0.57) and were not correlated with any of the environmental variables (all *p* > 0.05). There were significant differences between sex and age groups in mass gain (χ2 = 10.49, df = 3, *p* = 0.015), mass gain speed (χ^2^ = 8.51, df = 3, *p* = 0.037), and mass at departure (F = 2.748, df = 3, *p* = 0.0498). The Wilcoxon Rank Sum test showed that old females had on average a lower mass gain and mass gain speed than all other age and sex groups, and the Tukey HSD test showed that old females had on average a lower mass at departure than young males (Table 2). No significant differences were found between sex and age groups in mass at arrival (F = 0.897, df = 3, *p* = 0.45).

**Figure 3:**
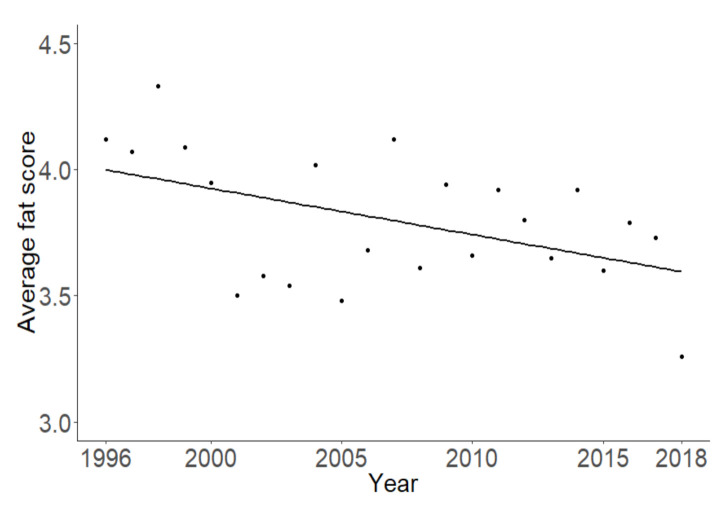
Average fat score of the Pied Flycatchers during stopover over the years.

The average fat score of the Pied Flycatchers at the study site decreased significantly over the years (t = −2.48; df = 1; *p* = 0.022, Figure 3).

**Figure 4:**
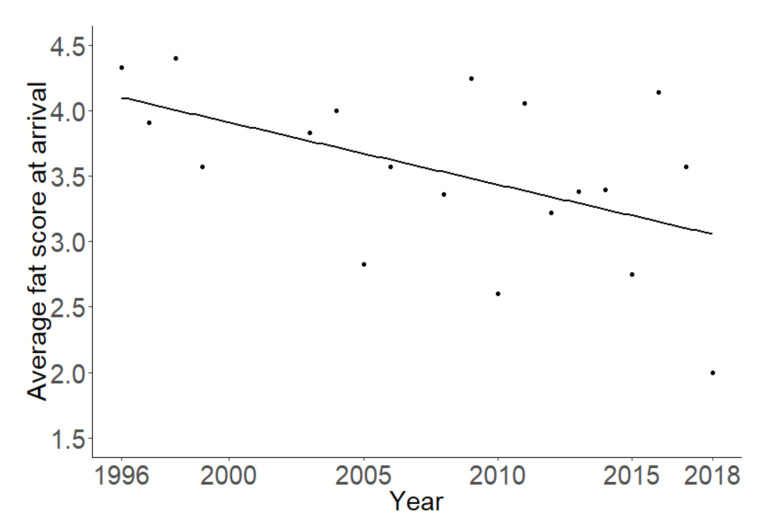
Average fat score of the Pied Flycatchers at arrival over the years.

**Figure 5:**
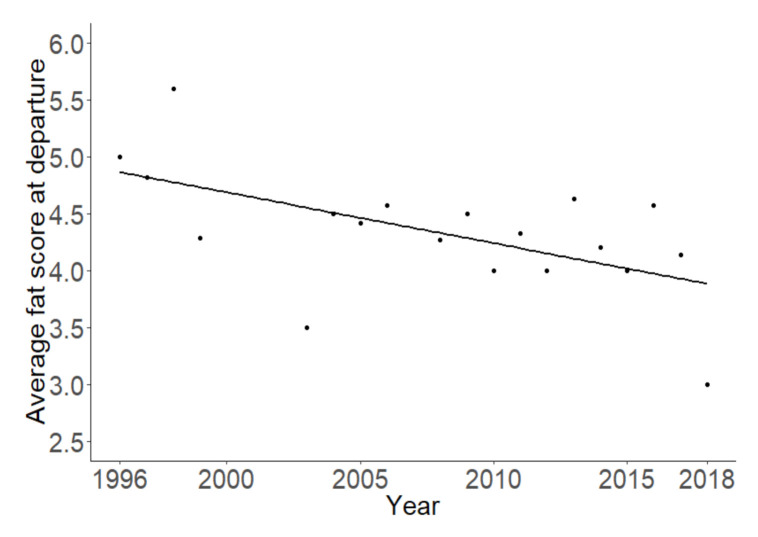
Average fat score of the Pied Flycatchers at departure over the years.

The amount of fat the Pied Flycatchers gained during their stay at the stopover site did not change significantly over the years (t = 0.19, df = 1, *p* = 0.85) and was not significantly correlated with any of the environmental variables (all *p* > 0.05). However, a decrease in both the amount of fat at arrival (t = −2.48; df = 1, *p* = 0.024, Figure 4) and the amount of fat at departure (t = −2.87; df = 1; *p* = 0.011, Figure 5) were found across the years. There were no differences in any of the fat score variables between sex and age groups.

## 4. Discussion

We did not find a significant change in the amount of individuals caught per year at the stopover site in southern Portugal, which was not in line with expectations, as Pied Flycatcher numbers are declining globally [30,31,45]. However, the territorial nature of the Pied Flycatcher, which is even present at stopover sites [28], may obscure possible population trends. Furthermore, Pied Flycatchers may be changing their migration routes so that a relatively larger part of the population is coming through southern Portugal, but no available data supports this theory. Local climatic variables did not affect the number of individuals caught per year either, which was in line with the expectations since the onset of the migration of birds is not determined by local climate conditions at stopover sites but rather by local climate conditions at the place of departure or on route to stopover sites [24,46]. However, we were unable to account for environmental conditions at the place of departure or on route, as it was not known where the birds at our study site originated from.

The arrival date of Pied Flycatchers at the stopover site did not advance over the years either, which is in contrast with findings from Cotton [24] and Jenni and Kéry [25], who investigated a wide array of migrant species (including the Pied Flycatcher in the study by Jenni and Kéry [25]) and found that long distance migrants wintering south of the Sahara had generally advanced their autumn migration because of changing climate in the Sahel region [25] and in a race to acquire a high quality wintering territory [47,48]. However, Pied Flycatchers did arrive earlier at the stopover site when the local average temperatures were higher, which may be related to higher temperatures across a larger part of the migration route or on the breeding grounds. Higher temperatures at the breeding ground have been shown to advance departure date [24]. Unfortunately, we could not take local climate situations at breeding grounds into account, as there were only few birds in our dataset that were ringed elsewhere during the breeding season and recaught at our study site.

The analysis of the flycatchers that were at least recaptured once during their stay showed that the minimum stopover duration of Pied Flycatchers increased over the years, but this was not related to any of the measured climate variables. This suggests that the increase in stopover duration over the years is, at least partially, explained by circumstances other than the measured weather conditions, such as a potential decrease in available food. A large decrease in insects has recently been recorded in Western Europe due to, amongst others, intensifying agriculture [49,50]. Although trends in insects have not been investigated on the Iberian Peninsula, some studies have investigated specific insect taxa. For example, a decline was found in butterflies [51] and dung beetles [52]. Furthermore, a recent review by Sánchez-Bayo and Wyckhuys [53] suggests that insect decline is a global phenomenon. Therefore, it is likely that a decline in insects is also present in southern Portugal, which could explain the need for the insectivorous Pied Flycatchers to reside at a stopover site for longer periods of time to gain sufficient fuel to continue their migration. However, data on insect abundance was not available at the study site; thus, unfortunately, we could not test this hypothesis in this study. Therefore, further research on the effects of insect decline on insectivorous songbird populations is recommended. The prolonged stopover duration during autumn migration we found is in contrast to findings by e.g., Tøttrup et al. [54], who found that migrants over time travelled quicker through Europe on route to their breeding grounds in northern Europe, which was also suggested by findings from Both et al. [23]. This stresses the fact that trends found for spring migration may not reflect patterns found for autumn migration and vice versa.

The average body mass of Pied Flycatchers caught at the stopover site did not change over the years, nor did mass gain, mass gain speed, and mass at departure. Mass itself is not the most reliable measure of energy reserves during migration, since it strongly depends on body size [55] and the amount of muscle [56], and it can show large diurnal variations [57]. However, the average fat score of Pied Flycatchers decreased over time, which may be a consequence of the potential worsening environmental conditions during migration or at the stopover site. Such worsening environmental conditions may be caused by a combination of drier conditions and an increase of forest fires in the region [32,33], which destroy an increasing amount of woodlands [34,35]. Although we did not have sufficient data for a reliable analysis with the amount of fires in the region, we recommend that forest fires are taken into account in future studies that investigate the effects of environmental changes on birds in areas that are sensitive to forest fires.

Pied Flycatchers both arrived with a lower average fat score and departed with a lower average fat score over the years. This supports the hypothesis that the migratory conditions for Pied Flycatchers have become worse over time: they arrive at the stopover site in a more energy-depleted condition and apparently cannot make up for this deficiency at the stopover site in southern Portugal either, even though they remain at the site longer. This is concerning, as from there, Pied Flycatchers start a long flight across the Atlantic Ocean toward Northern Africa [29] and are expected to need a certain amount of fat to successfully complete this flight [58]. If the average fat score at departure is decreasing, it is possible that fewer birds are able to complete the crossing to Africa.

The possibility that trends in the body condition of Pied Flycatchers occurred because of different people ringing and measuring birds over time cannot be omitted. However, there were in total 71 people taking measurements over the years, so we think it is unlikely that observed trends were largely caused by differences in measurements taken by different people. Unfortunately, we could not test for this, as there was no one single person taking measurements throughout the whole period. In addition, measurements of one person may not be consistent over time, either.

## 5. Conclusions

This study is one of the first studies to use a long-term ringing database to investigate trends in stopover behavior in migratory songbirds and to link these trends to local climate conditions. In conclusion, our study cannot confirm that Pied Flycatchers advanced their departure from breeding grounds, which has been found for other long-distance migrant passerines [24,25], since they did not advance the arrival at our study site (based on the first five percent of birds caught). They did not depart from the stopover site earlier or later over the years, either (based on the last five percent of birds caught). However, birds did arrive at and leave the stopover site with a lower fat score, and they spend more time at the stopover site. These findings may indicate worsening environmental conditions, such as food supply, during their migration to the stopover site as well as at the stopover site. The presence of environmental constraints during Pied Flycatcher migration has also been suggested [46]. Prolonged stopover duration and departure with a lower amount of fat than previously may eventually lead to decreased survival during onwards migration and delayed arrival at wintering grounds, as the migration speed of birds has been shown to depend mostly on stopover duration instead of on flying speed [59,60]. However, it is clear that variables other than local climate conditions also play a large part in explaining changing migration patterns and body condition at stopover sites. One factor that is also likely to be important is the global decrease in insect numbers [53]. The impact of this insect decline on songbirds still requires further study. This study strengthens the hypothesis that long-distance migratory songbirds are under threat [15] and suggests that the decline in migratory songbirds may not just be due to a phenological mismatch at the breeding grounds [31] but also due to worsening conditions during migration and at stopover sites. This should be considered when drawing up conservation plans. Further studies on the causes of these worsening conditions during migration that analyzes data from multiple species and other ringing stations at stopover sites are recommended in order to increase our understanding of the concerning decline in long-distance migratory songbirds.

## Figures and Tables

**Table 1:** All variables used in the analyses. Pied Flycatcher-related data were obtained from the A Rocha Portugal bird ringing database, and local climate variables were obtained from the weather station at the grounds of A Rocha Portugal.

Type	Variable
Migration strategy	Annual total amount of individuals captured
	Average arrival date at the study site
	Average departure date from the study site
	Average minimum stopover duration of recaptured individuals
Body condition	Average mass in grams
	Average fat score
	Average mass in grams at arrival ^1^
	Average mass in grams at departure ^1^
	Average fat score at arrival ^1^
	Average fat score at departure ^1^
	Average mass gain in grams ^1^
	Average gain in fat score ^1^
	Average mass gain speed in grams per day ^1^
	Average speed of gain in fat score per day ^1^
Local climate	Average temperature during the autumn migration period
	Total precipitation during the autumn migration period

^1^ Based on individuals that were recaptured at least once during their stay.

**Table 2:** The average mass gain and mass gain speed for each of the four age groups.

Group	N	Average Mass Gain (g)	Average Mass Gain Speed (g/day)	Average Mass at Departure (g)
Young males	25	2.03	0.47	16.50
Young females	20	1.75	0.48	15.55
Old males	8	1.90	0.49	15.85
Old females	17	0.54	0.15	15.08
